# Cell-extrinsic requirement for sulfate in regulating hippocampal neurogenesis

**DOI:** 10.1242/bio.053132

**Published:** 2020-07-31

**Authors:** Zhe Zhang, Dhanisha Jhaveri, Sazia Sharmin, Tracey J. Harvey, Paul A. Dawson, Michael Piper, David G. Simmons

**Affiliations:** 1 School of Biomedical Sciences, The University of Queensland, Brisbane, 4072, Australia; 2 Mater Research Institute, The University of Queensland, TRI Building, Woolloongabba, Brisbane, 4102, Australia; 3 Queensland Brain Institute, The University of Queensland, Brisbane, 4072, Australia

**Keywords:** Neurogenesis, Postnatal development, *Slc13a4*, Subgranular zone (SGZ), Sulfate

## Abstract

Sulfate is a key anion required for a range of physiological functions within the brain. These include sulfonation of extracellular proteoglycans to facilitate local growth factor binding and to regulate the shape of morphogen gradients during development. We have previously shown that mice lacking one allele of the sulfate transporter *Slc13a4* exhibit reduced sulfate transport into the brain, deficits in social behaviour, reduced performance in learning and memory tasks, and abnormal neurogenesis within the ventricular/subventricular zone lining the lateral ventricles. However, whether these mice have deficits in hippocampal neurogenesis was not addressed. Here, we demonstrate that adult *Slc13a4^+/−^* mice have increased neurogenesis within the subgranular zone (SGZ) of the hippocampal dentate gyrus, with elevated numbers of neural progenitor cells and intermediate progenitors. In contrast, by 12 months of age there were reduced numbers of neural stem cells in the SGZ of heterozygous mice. Importantly, we did not observe any changes in proliferation when we isolated and cultured progenitors *in vitro* in neurosphere assays, suggestive of a cell-extrinsic requirement for sulfate in regulating hippocampal neurogenesis. Collectively, these data demonstrate a requirement for sulfate transport during postnatal brain development to ensure normal adult hippocampal neurogenesis.

## Abstract (precis)

**Summary Statement**: Heterozygosity for the brain sulfate transporter *Slc13a4* during postnatal development results in life-long abnormal hippocampal neurogenesis.

## INTRODUCTION

Sulfonation is an essential, yet underappreciated, physiological process that regulates the function of a diverse array of endogenous molecules, and which plays key roles in cell–cell communication, growth and development. In the brain, important sulfonated substrates include neurosteroids, neurotransmitters, sulfogycolipids such as sphingolipids, and extracellular matrix molecules like heparan, chondroitin and dermatan sulfate proteoglycans (Strott, 2002), which are critical for brain development and function. Sulfonation of steroids, peptide hormones and neurotransmitters can alter their bioactivity through changes in receptor interaction, solubility or half-life, and typically, but not always, inhibits their bioactivity (Strott, 2002). On the other hand, the precise pattern of sulfonation on the glycosaminoglycan side chains of more structurally complex extracellular matrix proteoglycans influences their molecular interactions within microenvironments, altering their functions in both subtle and profound ways (Bülow and Hobert, 2006). This is particularly evident in the brain, where the sulfonation patterns on heparan, chondroitin and dermatan sulfates encode functional information and are known to effect important growth-factor binding and signalling within neural stem cell niches, influencing neural progenitor proliferation and differentiation (Bian et al., 2011; Purushothaman et al., 2012; Sirko et al., 2010). A recent example that has attracted increasing attention is the role of the sulfonation pattern of chondroitin sulfate within perineuronal nets (PNNs) and the regulation of critical periods of neurodevelopment. An increase in the C4/C6 sulfonation pattern of chondroitin sulfate leads to the termination of the critical period for ocular dominance in the mouse cortex, while artificially maintaining a low C4/C6 sulfonation pattern interferes with PNN formation and results in persistent cortical plasticity (Miyata et al., 2012).

While the temporal and spatial expression of substrate-specific sulfotransferases drives the precise sulfonation pattern of a substrate produced by a given cell or tissue, changing the availability of sulfate can also impact the output of cellular sulfonation reactions. Membrane sulfate transporters control sulfate availability both at the cellular and organ level (Dawson, 2011), and a number of sulfate transporters are known to be expressed in the brain (Dahlin et al., 2009; Ho et al., 2012; Zhang et al., 2019), where they presumably modulate the availability of sulfate. The sulfate concentration of cerebrospinal fluid is significantly lower than in plasma, suggesting a tight regulation of sulfate availability within the brain is important for normal brain development and function (Cole and Evrovski, 2000; Cole et al., 1982). Recently, our group demonstrated that a heterozygous loss of the sulfate transporter *Slc13a4* resulted in a reduction in the uptake of radio-labelled sulfate into the brain, as well as significant impairments in adult social behaviours and memory (Zhang et al., 2019). Moreover, *Slc13a4* heterozygosity was associated with prolonged alterations in the proliferation of neural stem cells within the adult ventricular/sub-ventricular (V-SVZ) stem cell niche. Conditional deletion experiments revealed that, with respect to these phenotypes, full biallelic expression of *Slc13a4* was only required during a narrow window of postnatal development, and loss of a single *Slc13a4* allele in adulthood did not result in the acquisition of either behavioural or cellular abnormalities (Zhang et al., 2019). Furthermore, treatment of heterozygous *Slc13a4* mice with N-acetylcysteine, a precursor for intracellular sulfate generation, within this postnatal window prevented the onset of proliferation defects within the adult V-SVZ.

Our previous study also revealed differences in hippocampal-dependent memory in mice lacking one allele of *Slc13a4*. Critically, the hippocampus is the other main source of ongoing neurogenesis within the rodent brain, with neural stem cells found within the subgranular zone (SGZ) of the hippocampal dentate gyrus. However, whether or not *Slc13a4*^+/−^ mice exhibit alterations to neural stem cell proliferation and differentiation within the SGZ remains unknown. The goal of this study was to analyse the functional significance of *Slc13a4* heterozygosity, and therefore reduced sulfate availability during postnatal development, on adult neurogenesis within the SGZ. We discovered that, as with the V-SVZ, proliferation and neurogenesis within the adult SGZ was increased, and that treatment with N-acetylcysteine postnatally could rescue these defects in adult mice. Moreover, using an *in vitro* neurosphere assay, we revealed that deficits in SGZ proliferation likely arise from a cell-extrinsic source.

## RESULTS

### Increased proliferation within the SGZ of adult *Slc13a4^+/−^* mice

**Fig. 1.**
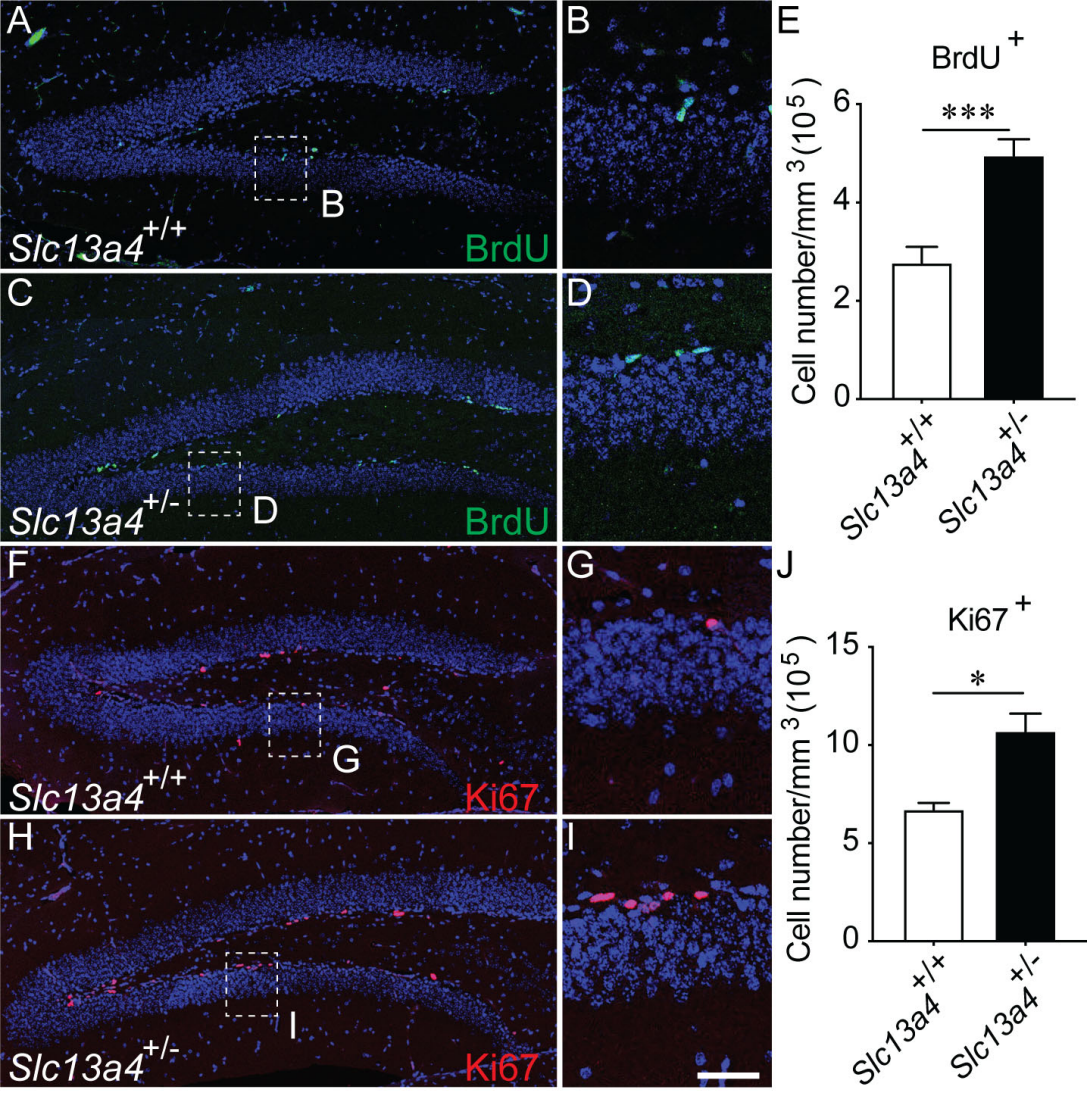
**Proliferation within the SGZ of *Slc13a4*^+/+^ and *Slc13a4*^+/−^ brains at 12 weeks.** Both BrdU injection/labelling (A–D) and Ki67 immunohistochemistry (F–I) revealed an increase in cell proliferation within the SGZ of *Slc13a4*^+/−^ brains compared with wild-type littermate controls (quantified in E and J, respectively) (*P*=0.035). B, D, G and I are higher magnification of dashed boxes in A, C, F and H, respectively. *n*=6 for BrdU quantification, *n*=3 for Ki67 quantification for each genotype. Mean±s.e., Student's *t*-test, *P<0.05. Scale bar: 200 µm in A, C, F and H, 25 µm in B, D, G and I.

We have previously shown that adult 12-week-old *Slc13a4^+/−^* mice exhibit neurogenic phenotypes within the V-SVZ, specifically an increase in the number of proliferating cells following acute administration of BrdU. This finding led to a number of subsequent questions. Was this effect also evident outside the V-SVZ? Did this increase in proliferation lead to elevated levels of transit amplifying cells and neurons? And, did elevated proliferation levels lead to a premature exhaustion of the progenitor pool in aged mice? To address these questions, we focused on the hippocampal SGZ. We administered BrdU to label proliferating cells within adult mice. Analysis of the dentate gyrus at 30 min following the last injection revealed significantly more BrdU-labelled cells within the SGZ of *Slc13a4^+/−^* mice in comparison to controls (Fig. 1A–E). Similarly, we observed significantly more Ki67-expressing cells in heterozygous mice (Fig. 1F–J).

**Fig. 2.**
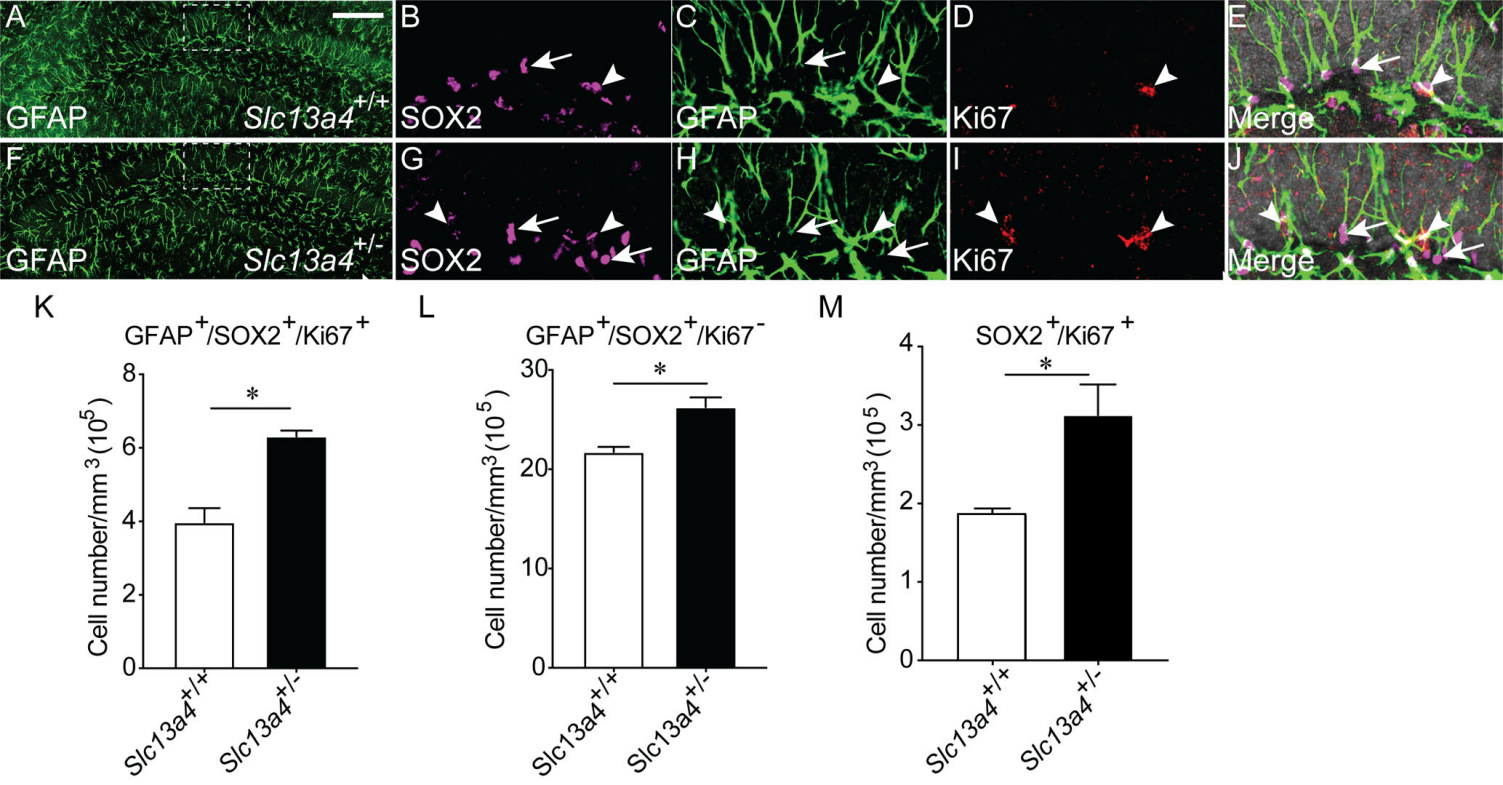
**Quantification of proliferating type 1 neural stem cells within the SGZ of *Slc13a4*^+/+^ and *Slc13a4*^+/−^ brains at 12 weeks.** (A) Representative lower magnification image from a wild-type SGZ labelled with GFAP, and higher magnification images labelled with (B) SOX2, (C) GFAP, (D) Ki67 or (E) merge. (F) A representative lower magnification image from a *Slc13a4*^+/−^ SGZ labelled with GFAP, and higher magnification images labelled with (G) SOX2, (H) GFAP, (I) Ki67 or (J) merge. (K) An increased number of proliferating type 1 neural stem cells (GFAP^+^/SOX2^+^/Ki67^+^; arrowheads in B–E, G–J) were observed in *Slc13a4*^+/−^ SGZ compared with wild-type controls. (L) An increased number of quiescent type 1 neural stem cells (GFAP^+^/SOX2^+^/Ki67^−^; arrows in B–E; G–J) were observed in *Slc13a4*^+/−^ SGZ compared with wild-type controls. (M) An increased number of SOX2^+^/Ki67^+^ cells were present in *Slc13a4*^+/−^ hilus compared with wild-type controls. *n*=3 for each genotype. Mean±s.e., Student's *t*-test, *P<0.05. Arrowheads highlight proliferating neural stem cells and arrows highlight quiescent neural stem cells. Scale bar: 200 µm in A and F, 25 µm in B, C, D, E, G, H, I and J.

**Fig. 3.**
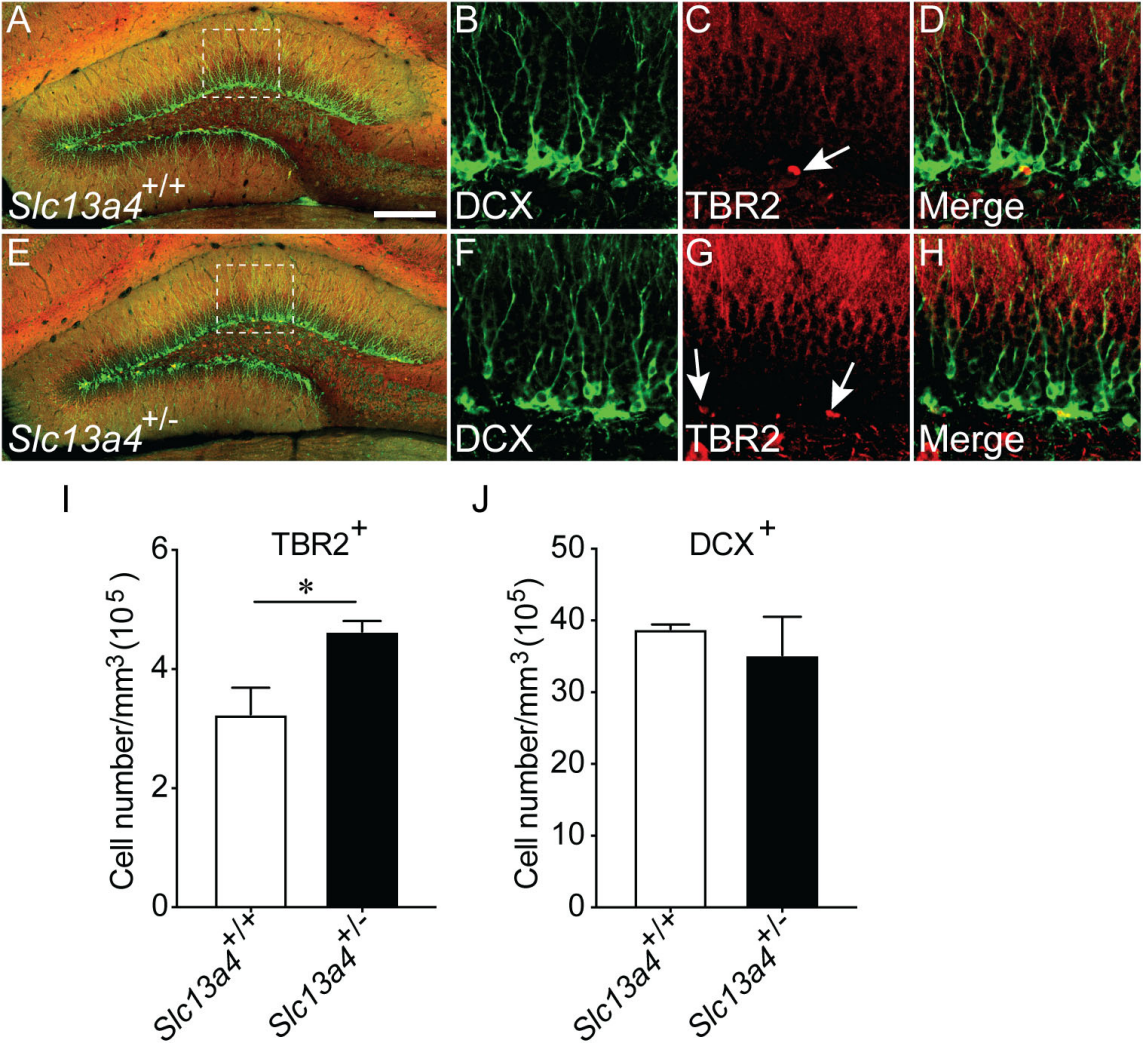
**Quantification of transient amplifying cells and neuroblasts within the SGZ of *Slc13a4*^+/+^ and *Slc13a4*^+/−^ brains at 12 weeks.** (A) Representative lower magnification image from a wild-type SGZ labelled with DCX and TBR2, and higher magnification images labelled with (B) DCX, (C) TBR2, (D) merge. (E) A representative lower magnification image from a *Slc13a4*^+/−^ SGZ labelled with DCX and TBR2, and higher magnification images labelled with (F) DCX, (G) TBR2 and (H) merge. (I) An increase in the number of transient amplifying cells (TBR2^+^) were observed in *Slc13a4*^+/−^ SGZ compared with wild-type controls. (J) No difference in the number of DCX^+^ neuroblasts was observed in *Slc13a4*^+/−^ SGZ compared with wild-type controls. *n*=3 for each genotype. Mean±s.e., Student's *t*-test, *P<0.05. Arrows highlight TBR2^+^ cells. Scale bar: 200 µm in A and E, 25 µm in B, C, D, F, G and H.

These data indicate an increased proliferation occurs within the SGZ neurogenic niche in the absence of one *Slc13a4* allele. To investigate this more thoroughly, we analysed the expression of cell-type-specific markers for cell populations within the SGZ. Neural stem cells within the SGZ express the cytosolic protein GFAP, as well as the nuclear protein SOX2. Staining with these markers, as well as with Ki67, enables the identification of quiescent (GFAP^+^/SOX2^+^/Ki67^−^) and activated (GFAP^+^/SOX2^+^/Ki67^+^) neural stem cells. Analysis of adult control and *Slc13a4^+/−^* mice revealed significantly more quiescent and proliferating neural stem cells within the SGZ of heterozygous mice (Fig. 2A–L). Moreover, we observed significantly more SOX2^+^/Ki67^+^ cells within the hilus of heterozygous mice (Fig. 2M), perhaps indicative of increased gliogenesis within this region of the *Slc13a4^+/−^* brain. Further from this, we also revealed significantly more cells expressing TBR2, a marker for transit-amplifying cells, but no increase in DCX-positive cells, a marker for neuroblasts, in the dentate gyrus of *Slc13a4^+/−^* mice in comparison to controls (Fig. 3A–J). Collectively, these data point to elevated proliferation of neural stem and progenitor cells, but not an increase in the generation of neuroblasts, in adult *Slc13a4^+/−^* mice.

**Fig. 4.**
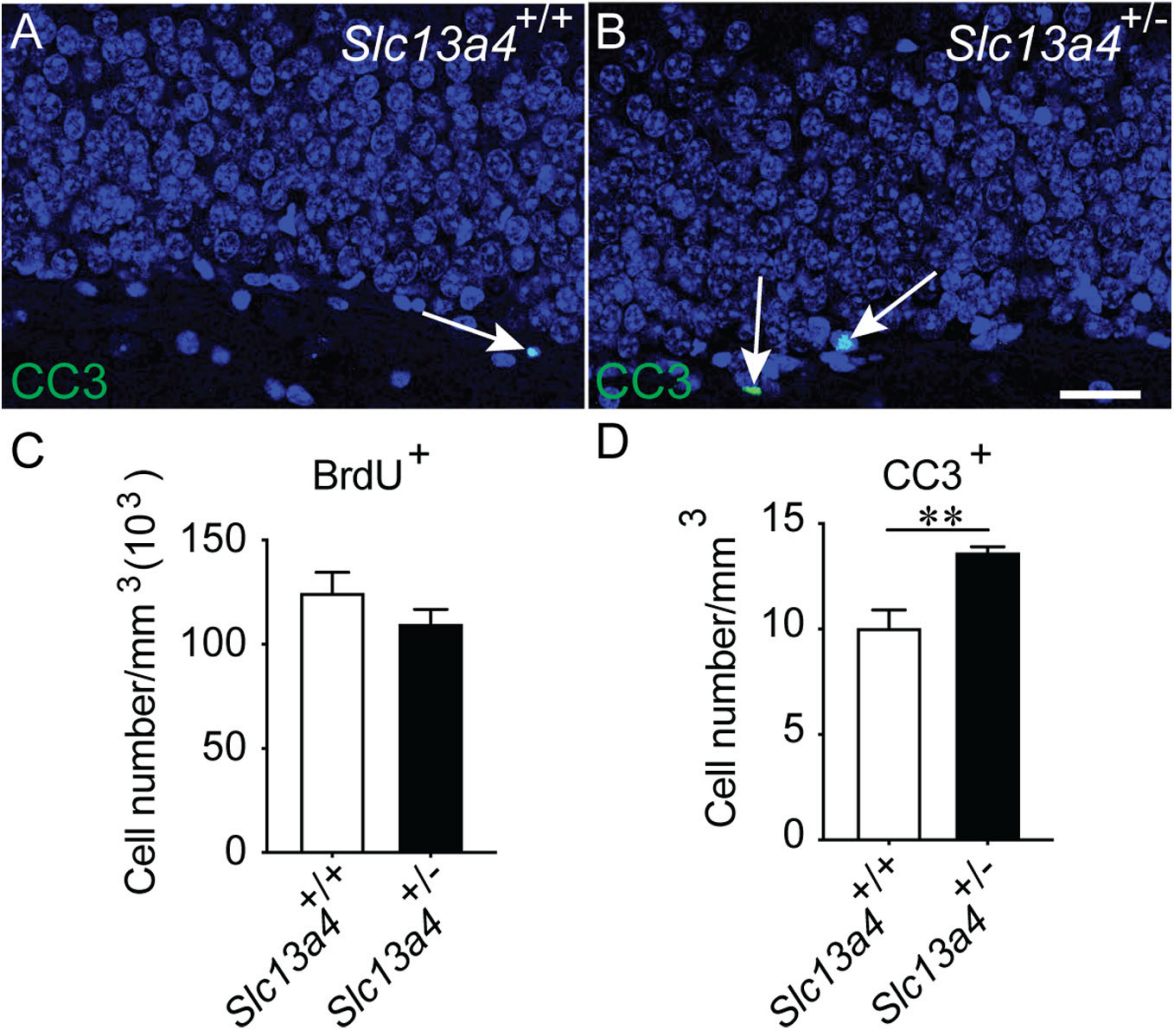
**Increased apoptosis in the *Slc13a4^+/−^* dentate gyrus.** Representative images of (A) wild-type and (B) *Slc13a4*^+/−^ dentate gyrus stained with DAPI (blue) and cleaved caspase 3 (CC3; green). CC3 was used as a marker for apoptosis and arrows indicate CC3-expressing cells. (C) The increase in neural progenitors from *Slc13a4*^+/−^ SGZ did not result in an increased number of BrdU-labelled cells in the dentate gyrus 28 days following BrdU pulse injection. (D) An increased number of CC3^+^ cells were detected in the *Slc13a4*^+/−^ dentate gyrus compared with wild-type littermates. *n*=5 for each genotype, Mean±s.e. Student's *t*-test, **P=0.0032. Scale bar: 25 µm.

Do these elevated levels of proliferation culminate in a larger number of neurons being generated? To assess this, we performed a 28-day pulse-chase experiment. Mice were given an intraperitoneal injection of BrdU to label proliferating cells and were analysed 28 days later. Interestingly, we observed similar numbers of BrdU-expressing cells within the granular zone of the dentate gyrus of control and *Slc13a4^+/−^* mice (Fig. 4A–C), consistent with the similar numbers of DCX-expressing cells within the dentate gyrus between genotypes (Fig. 3J). A similar result was seen when we analysed the olfactory bulbs as a proxy for neurogenesis within the V-SVZ (data not shown). This suggests that the increased proliferation we see in heterozygous mice does not culminate in elevated levels of newborn neurons, a finding supported by the higher levels of apoptosis within heterozygous mice in comparison to controls (Fig. 4D).

### Reduced neural stem cell number within the SGZ of aged *Slc13a4^+/−^* mice

**Fig. 5.**
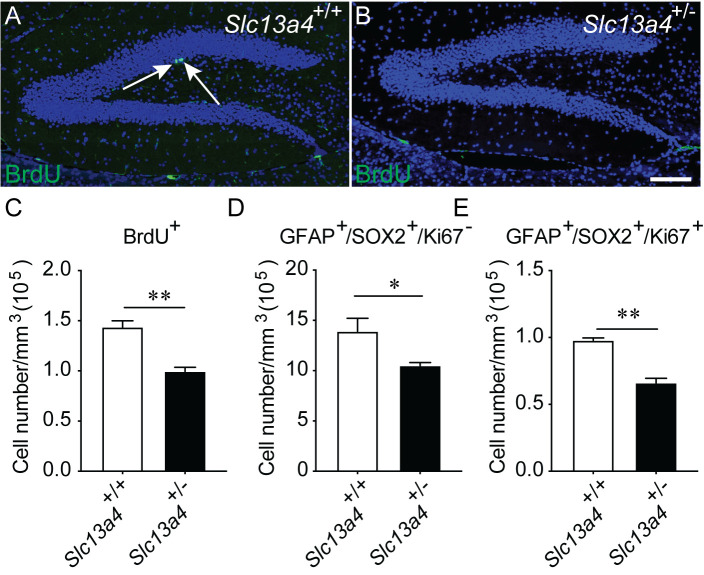
**BrdU-labelled cell proliferation in the SGZ of 12-month-old mice.** Representative images of the dentate gyrus of (A) *Slc13a4*^+/+^ and (B) *Slc13a4*^+/−^ mice. Arrows indicate BrdU^+^ cells (green). The number of BrdU^+^ cells in the 12-month-old *Slc13a4*^+/−^ SGZ was significantly less than that in the *Slc13a4*^+/+^ SGZ (C). (D) A significant decrease in quiescent neural stem cells (GFAP^+^/SOX2^+^/Ki67^−^) within SGZ was observed in *Slc13a4*^+/−^ mice compared with controls. (E) Similarly, there was a significant decrease in proliferating neural stem cells (GFAP^+^/SOX2^+^/Ki67^+^) in *Slc13a4*^+/−^ mice compared with controls. *n*=3 per genotype. Mean±s.e., Student's *t*-test, *P<0.05, **P<0.01. Scale bar: 200 µm.

Precocious stem cell division within the SGZ could potentially lead to reductions in the stem cell pool over the course of aging. To assess this, we performed an acute BrdU pulse on aged wild-type control and *Slc13a4^+/−^* mice at 12 months of age. Consistent with this hypothesis, assessment of proliferation at this time point revealed a significant age-related decline in BrdU^+^ cells in the brains of 12-month-old *Slc13a4*^+/+^ and *Slc13a4*^+/−^ mice compared with 12-week-old mice (data not shown). Interestingly, there were significantly fewer BrdU labelled cells in the SGZ of 12-month-old heterozygous mice than 12-month-old wild-type controls (Fig. 5A–C). In line with this, we also observed significantly fewer quiescent and proliferating neural stem cells in the SGZ of older *Slc13a4^+/−^* mice (Fig. 5D,E). These data suggest that the early increases we observed in proliferation within the SGZ may have long-term ramifications for stem-cell biology within the aged hippocampus.

### Postnatal N-acetylcysteine treatment rescues elevated proliferation seen within the SGZ of *Slc13a4^+/−^* mice

**Fig. 6.**
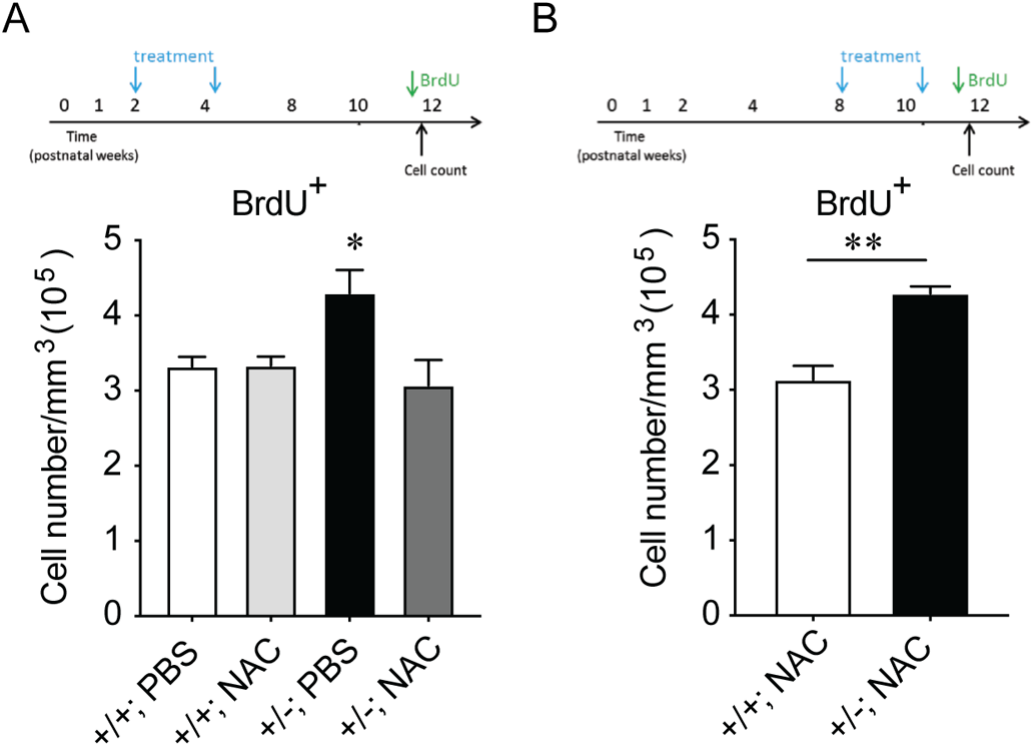
**Cellular proliferation in *Slc13a4*^+/+^ and *Slc13a4*^+/−^ adult SGZ after administration of N-acetylcysteine between postnatal days 14–30.** (A) N-acetylcysteine (150 mg/kg) or PBS was administered to *Slc13a4*^+/+^ and *Slc13a4*^+/−^ mice from P14–P30. BrdU injections were performed at 12 weeks of age. *Slc13a4*^+/−^ PBS-treated mice displayed increased BrdU^+^ cells within the SGZ compared to all other groups. Importantly, N-acetylcysteine administered *Slc13a4*^+/−^ mice did not display an increase in BrdU^+^ cells in the SGZ. One-way ANOVA were used followed by Dunnett's *post hoc* test to determine statistical significance. (B) N-acetylcysteine (150 mg/kg) or PBS was administered to *Slc13a4*^+/+^ and *Slc13a4*^+/−^ mice at 8 weeks of age (P56) for 16 days. BrdU injections were performed after the final injection. *Slc13a4*^+/−^ +NAC mice still showed increased cell proliferation within the SGZ compared to the wild-type +NAC mice (*P*=0.0050). *n*=3 per genotype. Mean±s.e., One-way ANOVA (A) and Student's *t*-test (B) were used for analysis, *P<0.05, **P<0.01. *n*=7–9 per genotype/treatment.

Treatment of *Slc13a4^+/−^* mice with N-acetylcysteine during postnatal development (P14-P30) rescues the social and memory deficits these mice normally exhibit in adulthood (Zhang et al., 2019). We next asked the question of whether N-acetylcysteine treatment also resolved the proliferation phenotype evident within the adult SGZ. Wild-type mice treated with N-acetylcysteine from P14–P30 did not have any significant difference in proliferation within the SGZ at 12-weeks of age in comparison to PBS-treated controls. Importantly, whereas *Slc13a4^+/−^* mice treated with PBS exhibited significantly more BrdU-labelled cells in comparison to PBS-treated controls, N-acetylcysteine-treated *Slc13a4^+/−^* mice had levels of proliferation equivalent to wild-type controls (Fig. 6A). As expected, treatment with N-acetylcysteine in adulthood did not resolve the SGZ proliferative effects (Fig. 6B), further suggesting the requirement for sulfate in the postnatal window is important for long-term brain function.

### Cell-extrinsic requirement for sulfate in neural progenitor cell proliferation

**Fig. 7.**
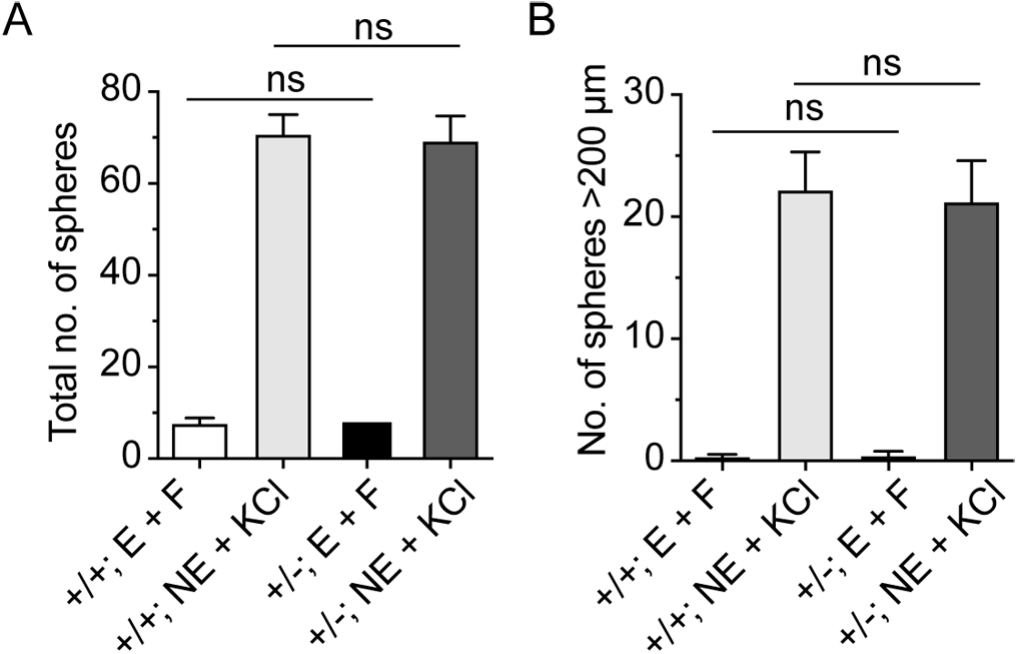
**The formation of neurospheres from *Slc13a4*^+/+^ and *Slc13a4*^+/−^ stem cell niches.** EGF (E) and bFGF (F) were used to stimulate the cell proliferation of isolated primary NSCs *in vitro*. No significant differences were observed in the total number of neurospheres (A) or in the number of large neurospheres (>200 µm, B) formed by isolated NSCs from the SGZ of either genotype (*n*=6 for each genotype). Similarly, when cells were stimulated with norepinephrine (NE) and potassium chloride (KCl) we saw a marked increase in neurosphere numbers (A) and size (B) in both genotypes. However, intergenotype comparisons revealed no difference between control and heterozygous mice. ns, not significant; *n*=6 per genotype; mean±s.e.

*Slc13a4* is predominantly expressed in the choroid plexus and pia mater, not within the neurogenic niches themselves (Zhang et al., 2019). This suggests that the phenotypes we observed with regard to proliferation within the V-SVZ and SGZ may be cell-extrinsic, possibly the result of altered sulfate availability within the niches. To assess this, we removed stem and progenitor cells from their niche, and cultured them *in vitro* in a primary neurosphere assay, in a controlled *milieu* with levels of sulfate that were equivalent between samples. We cultured cells under basal conditions (grown with EGF or FGF alone), or stimulated them with norepinephrine and KCl, which has been shown previously to activate proliferation in quiescent progenitor cells (Deleyrolle and Reynolds, 2009; Jhaveri et al., 2015). In either case, we saw no significant differences in either neurosphere number, or size, indicating that there were no cell-intrinsic deficits in neural progenitor cell proliferation in cells isolated from *Slc13a4^+/−^* mice (Fig. 7). This is consistent with our hypothesis for a cell-extrinsic mechanism underlying the proliferation defects we observed in mice lacking one allele of *Slc13a4^+/−^*.

## DISCUSSION

Mice heterozygous for *Slc13a4*, a gene encoding a sulfate transporter highly expressed in the choroid plexus and pia mater, display an approximate 50% reduction in the uptake of radio-labelled sulfate into the brain (Zhang et al., 2019), indicating this transporter may play an important role in regulating CSF and brain sulfate levels. We previously reported that loss of one allele of *Slc13a4* results in the onset of abnormal social behaviours, memory deficits and increased proliferation in the V-SVZ neurogenic niche of adult *Slc13a4*^+/−^ mice (Zhang et al., 2019). Given the role of the hippocampus and neurogenesis within the SGZ in learning and memory (Gonçalves et al., 2016), we investigated whether there was also a neurogenic phenotype in the SGZ niche in *Slc13a4*^+/−^ mice.

Akin to what we previously observed in the V-SVZ (Zhang et al., 2019), we also detected increased proliferation (BrdU uptake and Ki67 expression) within the SGZ neurogenic niche of adult *Slc13a4*^+/−^ brains. This increased proliferation included both increased numbers of neural stem cells (GFAP^+^/SOX2^+^) and transient amplifying cells (TBR2^+^), but not DCX^+^ neuroblasts. Increased apoptosis in the SGZ would suggest that the excess proliferation in *Slc13a4*^+/−^ brains is not translated into mature integrated neurons but rather cell loss. Indeed, a 28-day post-chase experiment failed to detect increased incorporation of newly born neurons in the dentate gyrus or in the olfactory bulb.

Neurogenesis declines as part of the normal process of aging, and is thought to contribute to age-related cognitive impairments (Kozareva et al., 2019). However, in *Slc13a4*^+/−^ mice this decline was exacerbated, suggesting the increased proliferation within the SGZ and V-SVZ in heterozygous mice may contribute to the exhaustion of the neural stem cell pool. It would be interesting to see if 12-month-old *Slc13a4*^+/−^ mice also displayed exaggerated age-related declines in memory and cognition. Nevertheless, it is now well established that both excessive proliferation within the neural stem cell niches in early adult life and exaggerated declines in neurogenesis in older animals result in cognitive and memory impairments, indicating that a delicate balance in the regulation of neurogenesis is required for optimal cognitive functions.

Importantly, establishment of the neural stem cell niches in postnatal development appears to be critical for life-long regulation of neural stem cell proliferation. We previously found that loss of one allele of *Slc13a4* at 8 weeks of age did not result in the onset of behavioural, memory or V-SVZ neurogenic phenotypes, whereas postnatal loss of *Slc13a4* activity did lead to those abnormal phenotypes (Zhang et al., 2019). In the current study, we found this to hold true for the SGZ also (Fig. S1). Moreover, supply of N-acetylcysteine during a critical postnatal window (P14–P30), which can circumvent sulfate transporters and be metabolized to free inorganic sulfate within tissues, can prevent the onset of SGZ neurogenic phenotypes in adult heterozygous animals. Therefore, there is a distinct developmental origin to the adult phenotypes observed in *Slc13a4*^+/−^ mice. But how is sulfate transport within the choroid plexus and pia mater connected to altered neurogenesis in the stem cell niches such as the V-SVZ and SGZ? Are there permanent alterations in the stem cells themselves, or structural changes within the niches that have long-lasting effects? We isolated neural stem cells from the SGZ and cultured them in a neurosphere assay (Deleyrolle and Reynolds, 2009; Jhaveri et al., 2015). In an *in vitro* environment where sulfate levels and the microenvironment are equivalent, neural stem cells isolated from *Slc13a4*^+/−^ brains were indistinguishable from neural stem cells isolated from wild-type animals in forming neurospheres. This suggests there are no cell-intrinsic differences in *Slc13a4*^+/−^ neural stem cells, but rather indicates that cell-extrinsic mechanisms likely underlie the proliferation defects observed *in vivo*.

Importantly, the sulfonation patterns of stem cell niche extracellular matrix proteoglycans can influence neurogenesis by regulating growth factor signalling; the most well-known being the regulation of fibroblast growth factor signalling by heparan sulfate proteoglycans within the V-SVZ (Mercier, 2016; Purushothaman et al., 2012; Yamada et al., 2017; Yu et al., 2017). Within the SGZ, chondroitin sulfate and dermatan sulfate also appear to play roles in regulating neurogenesis as well (Li et al., 2019; Yamada et al., 2018). It is possible that impaired sulfate availability during the critical postnatal developmental window, when the demand for sulfate is high, alters the patterning of ECM proteoglycans laid down in the neural stem cell niches. The consequences of altered sulfonation patterns on chondroitin sulfate laid down during critical postnatal periods in the brain can have lasting consequences, as seen when chondroitin 6-sulfotransferase (C6ST1) is overexpressed (Miyata et al., 2012). Overexpression of C6ST1 in the brain impairs perineuronal net formation and alters cortical plasticity. It will be important to ascertain whether the sulfonation patterns of chondroitin sulfate and dermatan sulfate are skewed in developing and adult *Slc13a4*^+/−^ SGZ mice, and whether this leads to the increased proliferation within this stem cell niche.

## MATERIALS AND METHODS

### Animals

All the experiments involving animals were performed with approval from the Animal Ethics Committee, University of Queensland. *Slc13a4^+/−^* males were bred with wild-type C57BL/6 females to generate *Slc13a4^+/+^* and *Slc13a4^+/−^* mice. UBC-Cre^ERT2^ mice were crossed with *Slc13a4^flx/+^* mice to generate *Slc13a4^+/+;^UBC-Cre^ERT2^* and *Slc13a4^+/flx;^UBC-Cre^ERT2^* mice.

### Genotyping

Genomic DNA was extracted from mouse toe samples using the KAPA mouse genotyping kit (KAPA Biosystems, USA). PCR was performed following the manufacturer's instructions. Primer sequences and PCR conditions for genotyping have been previously reported (Zhang et al., 2019).

### Drug treatment

BrdU (Sigma-Aldrich) (50 mg/kg/injection) was administered to mice at 10–14 weeks of age via intraperitoneal (IP) injection. BrdU was administered to animals five times on the day of the experiment, 2 hours apart. 30 min after the final injection, mice were euthanised. For BrdU pulse experiment, mice were injected four times with BrdU, 2 hours apart. Brains were harvested 28 days later to examine the migration of BrdU labelled cells.

Tamoxifen (Sigma-Aldrich) was dissolved in 1% ethanol and sesame oil as a 10 mg/ml stock solution. For adult *Slc13a4^+/flx^;UBC-Cre^ERT2^* and *Slc13a4^+/flx^* mice, 100 mg/kg tamoxifen was administered by IP for 5 consecutive days. For neonatal animals, 50 mg/kg tamoxifen was injected by IP for 4 consecutive days.

For NAC administration, 150 mg/kg NAC was administered to mice IP once per day for 16 days, from P14–P30 or from 8–10 weeks.

### Immunofluorescence

Paraffin sections were deparaffinised and rehydrated with ddH2O. Antigen retrieval was performed in an antigen retriever (Antigen Retriever 2100; Electron Microscopy Sciences, Australia) with citric acid buffer (pH6.0). Slides were then cooled to room temperature and washed with PBS. Slides were blocked with 10% donkey serum for 1 h, followed by primary antibody (BrdU, 1:200, DSHB; GFAP, 1:500, Abcam; DCX1:500, DB; SOX2, 1:200; Ki67, 1:1000, Abcam; Cleaved caspase 3, 1:200, CST; TBR2, 1:500, Abcam) incubation at 4°C overnight. Slides were washed with PBST for 5 min three times on the second day and then incubated with secondary antibody for 1 h at room temperature, followed by 5 min DAPI counterstain. Slides were mounted with 70% glycerol.

### Imaging

Images were captured on a Leica DMi8 confocal microscopy or Olympus BX61 fluoresce microscope and analysed by ImageJ. Every twelfth 8 µm sagittal serial section was collected from lateral 0.60 mm to lateral 1.80 mm (relative to Bregma) to quantify BrdU^+^, Ki67^+^cell numbers, cleaved caspase 3^+^ cells and neural progenitors. Samples were de-identified and researcher was blind to genotype when cell counting was performed.

### Adult neurosphere culture

10–12-week-old female mice were euthanised by cervical dislocation and brains were dissected into ice-cold Hank's essential medium. Further micro-dissection of the hippocampus, as well as primary neurosphere cultures, were performed following previously published protocols (Jhaveri et al., 2015).

### Statistical analysis

Quantitative data is presented as mean±s.e. of the mean, analysed and graphed using GraphPad Prism 6.0. Student's *t*-test was performed to analyse data between two groups. Welch's correction was performed if significant difference was evident in *F*-test. *P*-values and sample sizes are listed in the figure legends for each individual experiment.
